# The type 2 inflammatory response favors recognition of tumor antigens by IgE in breast cancer

**DOI:** 10.1002/cnr2.2002

**Published:** 2024-02-22

**Authors:** Espiridión Ramos‐Martinez, Francisco Javier García‐Vazquez, Ramcés Falfán‐Valencia, Jorge Rojas‐Serrano, Ana Alfaro‐Cruz, Marcela Pérez‐Villaseñor, Gerardo Aristi‐Urista, Jesús Pérez‐Hernández, Rosario López‐Vancell, Andrea Velasco‐Medina, Guillermo Velázquez‐Sámano

**Affiliations:** ^1^ Unidad de Medicina Experimental, Facultad de Medicina Universidad Nacional Autónoma de México Mexico city Mexico; ^2^ Departamento de Análisis Clínicos y Estudios Especiales Instituto Nacional de Pediatría, Laboratorio de Inmunogenética Molecular Mexico city Mexico; ^3^ HLA Laboratory Instituto Nacional de Enfermedades Respiratorias Ismael Cosío Villegas Mexico city Mexico; ^4^ Unidad de Enfermedades del Intersticio Pulmonar y Reumatología, Instituto Nacional de Enfermedades Respiratorias, “Ismael Cosío Villegas” Mexico city Mexico; ^5^ Patología Quirúrgica, Servicio de Anatomía Patológica, Hospital General de México, “Dr. Eduardo Liceaga” Mexico City Mexico; ^6^ Servicio de Anatomía Patológica. Hospital Regional de Alta Especialidad del Bajío León Mexico; ^7^ Servicio de Alergia e Inmunología Clínica, Hospital General de México, “Dr. Eduardo Liceaga” Mexico City Mexico

**Keywords:** breast, cancer, IgE, inflammatory response

## Abstract

**Background:**

Several studies describe an inverse statistical relationship between the presence of an allergy and development of cancer. However, the immunological mechanism involved in the relationship between these two degenerative diseases has not been explored.

**Aims:**

The main objective of this study was to explore the possibility that the lymphocyte T helper (Th) 2 response, a characteristic of allergy, induces recognition of tumor antigens.

**Methods and results:**

Patients with a clinical diagnosis of breast ductal carcinoma were included. Histopathological markers related to proliferation of tumor cells were determined (Her‐2‐neu, Ki‐67, estrogen receptor, and progesterone receptor). IHC was performed using IgE antibodies purified from an allergy patient and from each biopsy donor patient. Serum concentrations of cytokines representative of Th1 and Th2 inflammatory responses were determined. A total of 14 patients with a confirmed diagnosis of breast ductal carcinoma were included. IHC performed on biopsies showed a weak response when using purified IgE antibodies from an allergy patient; however, IHC using the IgE of each patient as the primary antibody showed an intense and highly specific signal. Serum concentrations of cytokines of the Th2 response, that is, IL‐4 (130.5 pg/mL (116–135 pg/mL)), IL‐5 (202 pg/mL (191–213 pg/mL)), and IL‐13 (105.5 pg/mL (98–117 pg/mL)), were significantly higher than those of the Th1 response, that is, IL‐6 (86 pg/mL (79–90 pg/mL)) and INF‐γ (93 pg/mL (79–99 pg/mL)).

**Conclusion:**

Purified IgE antibodies specifically recognize tumor cells in breast ductal carcinoma.

## 1 INTRODUCTION

It is well known that development of cancer and its response to treatment are phenomena regulated by inflammation; this immunological mechanism can regulate malignant cell growth or favor proliferation in an organism.^1^ In a classic tumorigenesis model, a single cell can serve as the origin of cancer. For a cell that constantly receives attacks of different types, the internal repair mechanisms break down, which is reflected in a series of somatic changes that promote cell transformation and dissemination of tumors, comprising a large number of cells that are not subjected to the mechanisms of regulation of growth and death by apoptosis.^2^ Indeed, is a close relationship between chronic inflammation and tumor development has been described. For example, this relationship has been demonstrated in colorectal cancer, for which Crohn's disease is a crucial predisposing factor.^3^ Despite the latter, the primary objective of the immune system is to distinguish between what is self and what is not self to discern through the activity of different receptors if what is foreign represents a risk and then develop a response focused on neutralizing and eliminating the threat. In various works, it has been determined that tumors are immunogenic since they trigger specific responses aimed at controlling the proliferation of tumor cells; however, it has also been documented that this immune response is nonresolving.^4^

It has been described that among the three main lineages of T helper lymphocytes (Th1, Th2, and Th17), those that differentiate as Th1 constitute the most remarkable presence in most types of cancer. These lymphocytes secrete interferon‐γ and assist cytotoxic lymphocytes and tumor‐fighting M1 macrophages,^1^ whereas the responses induced by Th2 and Th17 in many cases are detrimental since they have been related to promoting growth, differentiation, and dispersion of tumor cells.^5^, ^6^ However, certain elements induced by these two lymphocyte polarizations might favor cancer resolution. For example, there is evidence that Th17 cells play a beneficial role in halting tumor progression by stimulating the activity of Th1 and effector cells.^7^, ^8^ In the specific case of the Th2 response, little information exists; nevertheless, several epidemiological studies have evaluated a potential inverse association between allergy and risk of malignancy. Hsiao et al., in a meta‐analysis, found an inverse relationship between the presence of allergy history and risk of head and neck cancer.^9^ On the other hand, Ajrouche et al. described an inverse relationship between the background of asthma and allergic rhinitis and development of leukemia, and this relationship may be altered in the presence of genetic polymorphisms of Th2 pathway cytokines.^10^

Th2 cells play an essential role in the host defense against helminthic pathogens and allergens by producing Th2 cytokines, such as interleukin‐4 (IL‐4), IL‐5, and IL‐13, to trigger inflammatory responses, as characterized by high production of IgE isotype antibodies.^11^ The nature of the biological relationship between cancer and allergy has interested epidemiology, oncology, and immunology researchers for several decades without, until now, documenting any immunological mechanism that justifies such statistical association.

An essential requirement for induction of any immune response is prior recognition of antigens by innate or adaptive receptors. With this in mind, we developed this work with the main objective of exploring the possibility that IgE antibodies, characteristic of the type 2 inflammatory response, recognize tumor cell antigens.

## 2 MATERIAL AND METHODS

### 2.1 Patients

Cancer patients from the Spaniard Sanatorium, a private hospital in Torreón, Coahuila, Mexico, were included in this study. The patients were enrolled between February and November 2022. The inclusion criteria included patients with a confirmed diagnosis of breast ductal carcinoma of legal age and signing informed consent about the scope and purposes of the study. The exclusion criteria were comorbidities, pregnancy, allergic history and chemotherapeutic or immunosuppressive treatments. As part of the diagnostic procedure for each patient, different antibody isotypes were determined using turbidimetry and nephelometry, and histological diagnosis was established through biopsy material embedded in a paraffin block to determine histopathological markers such as Her‐2‐neu, Ki67, estrogen receptors, and progesterone receptors. Each marker in the biopsy specimen was quantified, and the averages of positive cells in 10 high‐power fields (equivalent to 400 magnifications) were compared. Estimation of the intensity of cell proliferation markers in IHC preparations was carried out according to the recommendations of The American Society of Clinical Oncology and the College of American Pathologists for Her‐2‐neu status. Specifically, 0 was considered a sample without observable staining, + with a weak signal in few cells, ++ with a weak to moderate complete staining in >10% of the tumor cells observed and +++ strong complete staining in >10% of tumor cells, always taking as reference a previous preparation with +++ of each marker.^12^ Two‐micron‐thick sections were obtained for IgE staining from the same paraffin block. In addition, a patient with a history of atopy and respiratory and skin allergies with IgE immunoglobulin levels at a diagnosis of more than 7000 IU/mL at the allergy and immunology service of the General Hospital of Mexico “Eduardo Liceaga” was selected to join the study as a blood donor for IgE purification. The local institutional review board approved the study protocol (approval code number: DI/19/309‐A/04/102). In all cases, the patients were informed of the objectives and scope of the study and signed informed consent to participate.

### 2.2 Blood samples

A 250 mL total blood sample was collected from the allergy patient with serum levels of 7000 IU of IgE; after centrifugation, the serum was separated and stored in 10 mL aliquots kept at −80°C until use in the purification of IgE antibodies. Additionally, eight mL of blood was obtained without anticoagulant from each breast cancer tumor tissue biopsy donor. The serum was separated by centrifugation and stored at −80°C until it was used to purify IgE antibodies.

### 2.3 IgE purification

IgE antibodies were purified from the serum of the allergy patient and 14 donors of breast tumor tissue. This process was performed using the CaptureSelect™ IgE Affinity Matrix system (Thermo Fisher Scientific). Briefly, the column was packed in Econo‐Column® Chromatography Columns, 2.5 × 10 cm and equilibrated with ten volumes of equilibration buffer (20 mM Tris, 150 mM NaCl, pH 7.5) at a flow of 150 cm/h. The sample was loaded on the column and incubated overnight at 4°C and then washed with ten volumes of equilibration buffer. The samples were eluted with five volumes of elution buffer (20 mM sodium citrate, pH 3.5), and the recovered antibodies were quantified using the Bradford Assay™ (Bio‐Rad). The concentration of the samples was adjusted to 1200 μg/mL in equilibration buffer and stored at −80°C until used for immunostaining.

### 2.4 Immunohistochemical (IHC) staining

For hematoxylin/eosin (H&E) staining and IHC, surgical biopsies were fixed in 3.7–4.0% formaldehyde buffered with phosphate buffer and embedded in tissue medium composed of purified paraffin and regulated molecular weight plastic polymers, with a melting point of 56°C spiked with DMSO. Cuts at 2 and 3 μm were made; the first ones were used for histopathological interpretation with H&E, and the second ones for IHC using the Biotin‐free protein detection system. Epitope Retrieval Solution ER 2 10X pH 9 (Novocastra Leica Biosystems Newcastle Ltd, United Kingdom) was used for epitope unmasking. To block the activity of endogenous peroxidase, the samples were treated with 0.9% hydrogen peroxide in an aqueous medium for 5 min. Three cuts of each sample were used for IHC markers. The sections were incubated for 45 min with purified IgE antibodies from each patient at a 1:50 dilution, with monoclonal anti‐major eosinophil protein antibody at a 1:25 dilution (BMK13; Chemicon International, Temecula, CA, USA) and CD117 rabbit monoclonal antibody clone EP10 (previously known as Y145) (Biocare Medical) at a 1:50 dilution. Subsequently, the histological sections were incubated with the conjugated anti‐IgE antibody HRP BondTM Polymer Refine Detection for 10 min (Leica Biosystems Newcastle Ltd, United Kingdom). To visualize the reaction, 3,3′‐diaminobenzidine‐H_2_O_2_ was used as a substrate (Biocare Medical CA USA) for 5 min, as monitored under a microscope. Contrast was achieved with Mayer's hematoxylin (Lillie's Modification) (ScyTek Laboratories, Inc. Logan, UT, USA) and 0.37 M ammonium hydroxide solution.

### 2.5 Quantification of cytokines

The immune status of the breast tumor tissue donors was analyzed by quantification of circulating cytokines in serum, in addition to comparison of these molecules with those found in a control group of seventeen clinically healthy women. The concentrations of IL‐4, IL‐5, IL‐6, IL‐10, IL‐13 and IFN‐γ (all from PeproTech™ México) were determined by ELISA. All procedures were performed following the manufacturer's recommendations.

### 2.6 Statistical analysis

We describe data as means + SDs or medians (IQR) according to the distribution of variables. To compare Th1 and Th2 cytokines among each patient, we used the paired t test or the Wilcoxon signed‐rank test according to the data distribution. All analyses were two‐sided; α was set at 5% unless otherwise specified. Statistical software Stata v. 14.2 was employed to perform the analysis.

## 3 RESULTS

### 3.1 Patients

Fourteen female patients were included in this study, all from the Mexican states of Coahuila and Durango in northern Mexico. Based on medical records, auscultation, and laboratory tests, all patients received a breast ductal carcinoma diagnosis by a specialist oncologist at the Spaniard Sanatorium in Torreon, Coahuila, Mexico. The included patients presented anthropometric data usually observed in the Mexican population, even with a body mass index (BMI) that shows overweight as a characteristic of this sample of patients. All patients had a history of being married, most with a history of having children and using contraceptives (Table 1). Eleven patients had only one tumor focus; one patient had two foci, and two patients had three tumor foci; in all cases, a biopsy sample was taken from only one focus.

**TABLE 1:** Primary biometric, demographic and clinical data of the 14 patients diagnosed with breast ductal carcinoma.

Variable	*n* = 14
Age, years old^b^	60.5 (51–68)
Height, m^b^	1.54 (1.5–1.58)
Weight, kg^b^	73.5 (67–76)
BMI^b^	29.77 (28.95–30.58)
Age of menarche^b^	12 (11–12)
Marital state	
Married^a^	11 (78%)
Widowed^a^	3 (22%)
Parity	
Nulliparous^a^	1 (7%)
Parous^a^	13 (93%)
Oral contraceptive history^a^	11 (78%)
Antibodies	
IgA (mg/dL)^b^	548.43 (487.21–645.34)
IgG (mg/dL)^b^	1441.03 (1356.22–1655.67)
IgM (mg/dL)^b^	168.43 (157.22–183.87)
IgE (IU/mL)^b^	246.55 (212.76–278.89)

^a^ Categorical variables are described with percentages.

^b^ Medians (IQR).

### 3.2 Histopathological markers

**FIGURE 1:**
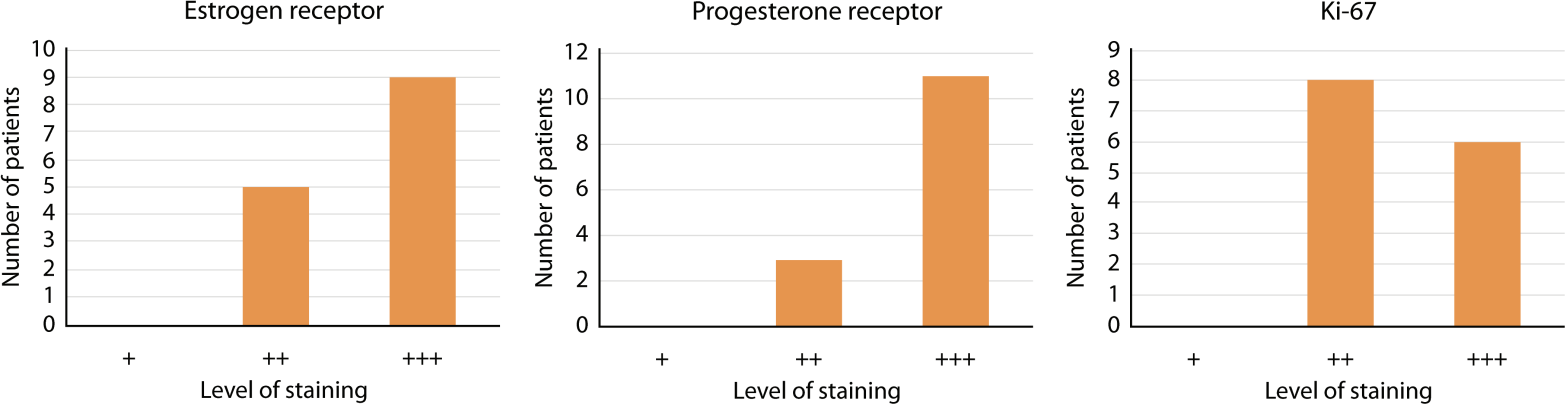
Number of patients with the intensity level of each tissue proliferation marker. Intensity, 0 was considered a sample without observable staining, + with weak signal in few cells, ++ with weak to moderate complete staining in >10% of the tumor cells observed and +++ strong complete staining in >10% of tumor cells.

As part of the diagnostic procedures for the biopsy of each patient, four histopathological markers were determined, all of them related to development of breast ductal carcinoma. IHC and interpretations were performed by an expert oncopathologist at Spaniard Hospital in Torreon, Mexico. The IHC results showed significantly different levels in the percentage of positive neoplastic cells for all markers. For Her‐2‐neu, samples from the 14 biopsy donor patients were negative. With respect to the other three proliferation markers, it was not possible to observe a pattern in their expression, as the intensity levels were highly heterogeneous. However, in all cases, ki‐67, progesterone receptor, and estrogen receptor were greater than ++ in intensity (Table 2). The intensity observed for each of the markers is shown in Figure 1. According to this determination, the breast ductal carcinoma presented by the biopsy donor patients was luminal A, with favorable prognosis.

**TABLE 2:** Intensity of cell proliferation markers in each of the patients.^a^

Patient	Her‐2‐nu	Ki‐67	Progesterone receptor	Estrogen receptor
A	0	++	+++	+++
B	0	+++	++	+++
C	0	+++	+++	+++
D	0	+++	+++	+++
E	0	+++	+++	+++
F	0	+++	+++	+++
G	0	++	+++	++
H	0	++	+++	+++
I	0	++	++	++
J	0	++	+++	++
K	0	+++	+++	++
L	0	++	+++	++
M	0	++	++	+++
N	0	++	+++	+++

^a^ According to the recommendations of The American Society of Clinical Oncology and the College of American Pathologists.

### 3.3 IHC with IgE in tumor biopsies

**FIGURE 2:**
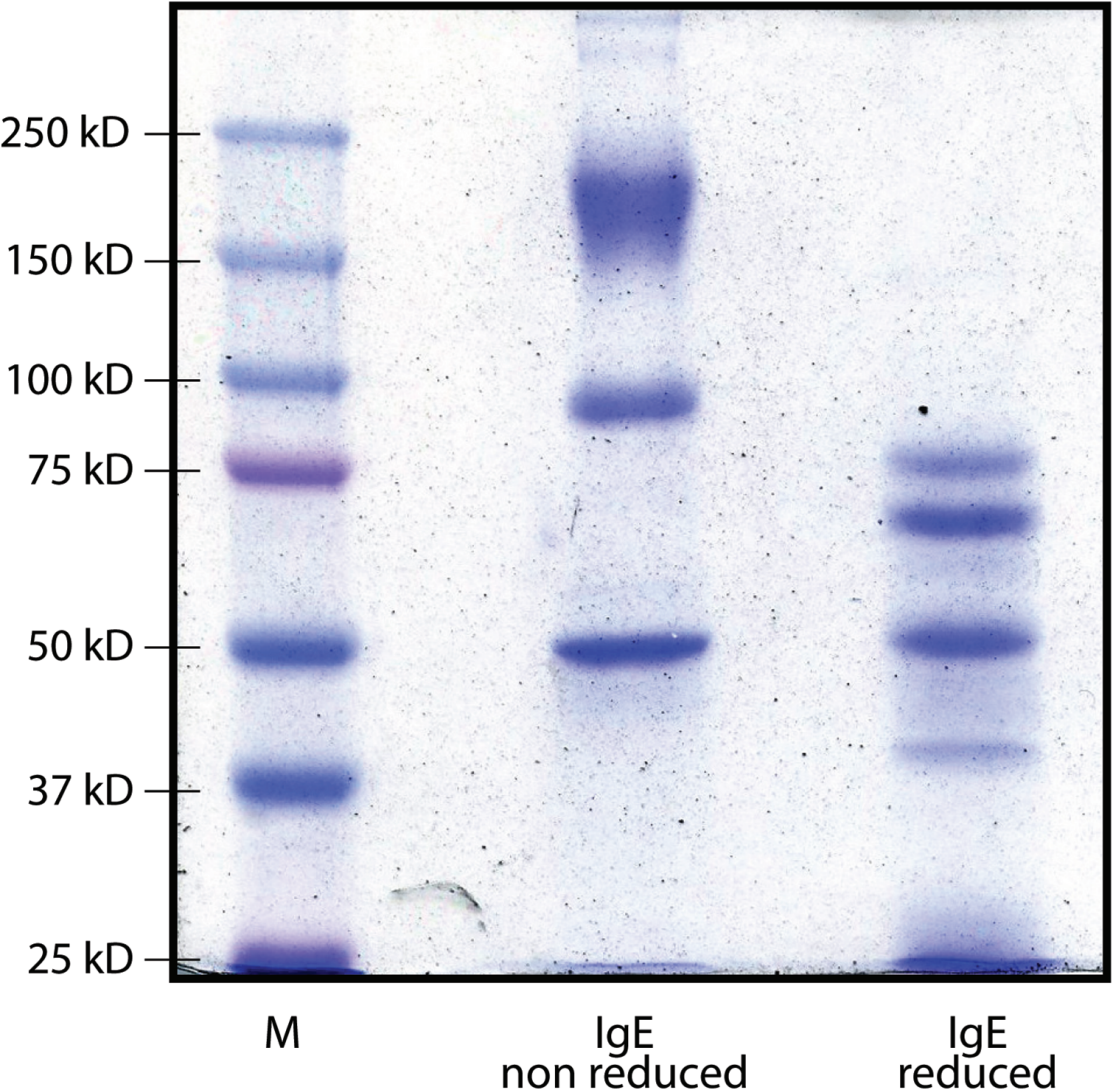
SDS–PAGE showing purified IgE from the allergy patient, a 7.5% gel was used. Electrophoresis was carried out under denaturing conditions, and the sample was assessed in a nonreduced and a reduced form with DTT. M = marker of molecular weight (Protein Dual Color Standard (Bio‐Rad™)).

**FIGURE 3:**
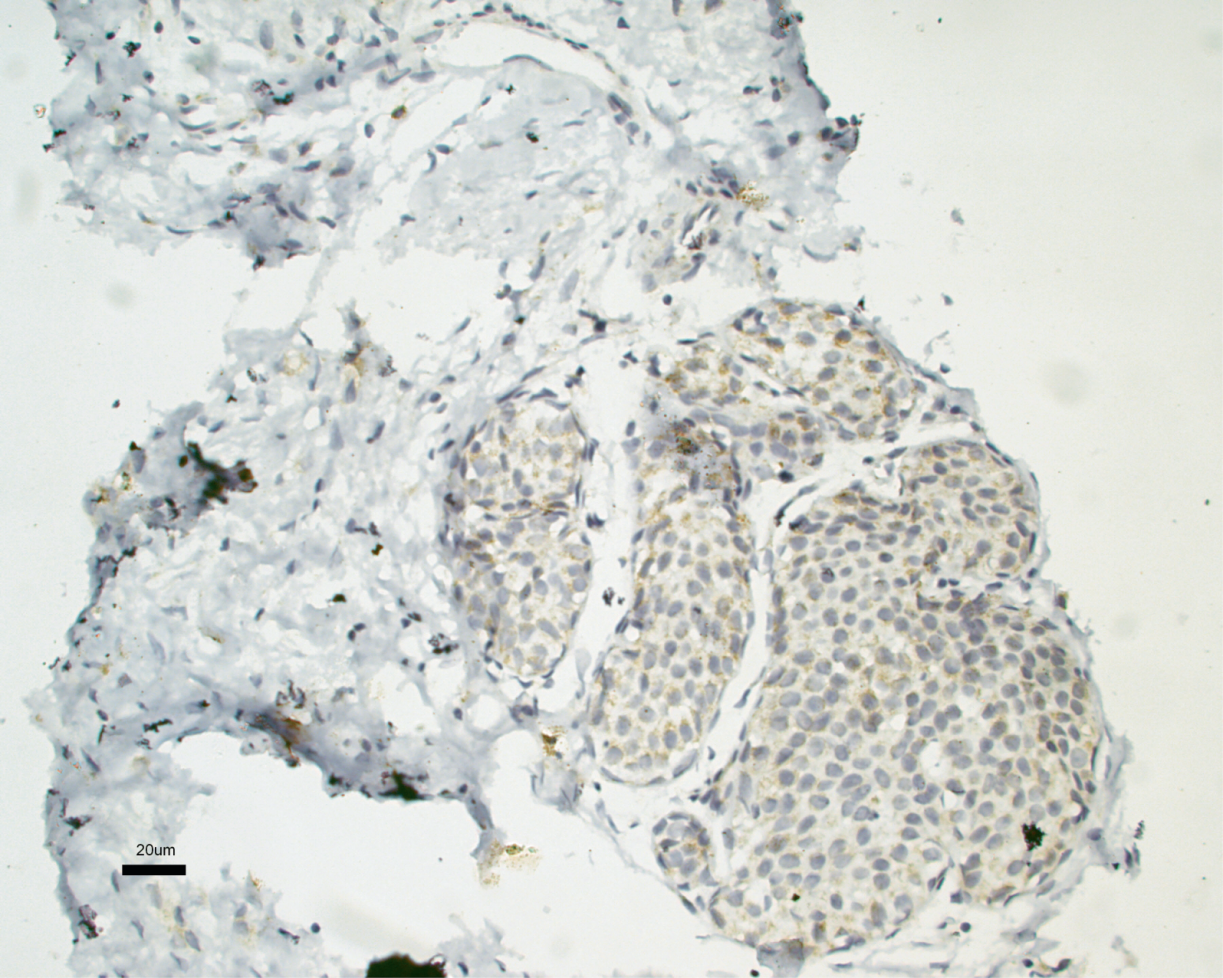
IHC photomicrograph of a breast ductal carcinoma biopsy. Purified IgE from a patient with respiratory allergy and atopic dermatitis was used as the primary antibody in the reaction.

**FIGURE 4:**
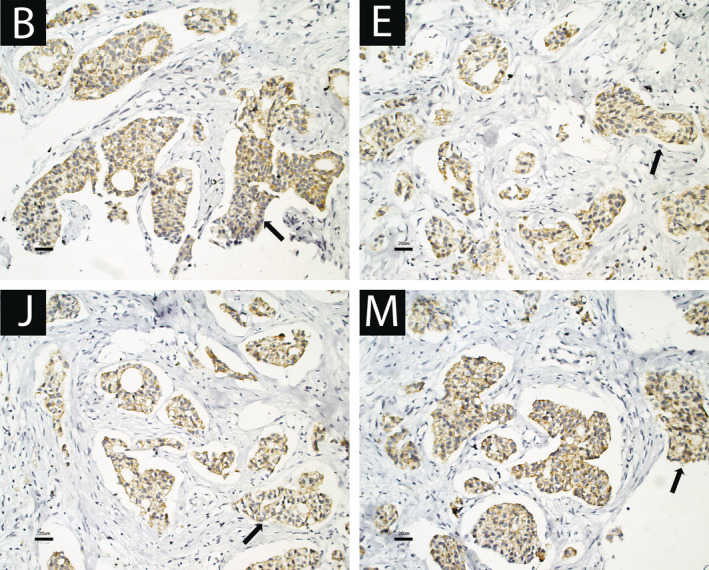
IHC photomicrograph of a breast ductal carcinoma biopsies. Purified IgE from each breast ductal carcinoma biopsy donor patient was used as the primary antibody in the reaction. Representative image of the findings for all patients. The letters in the boxes correspond to the patient identification, and the histological markers in each patient are described in Table 2. Arrows show immunostaining with moderate intensity and a diffuse and complete membranous pattern.

IgE antibodies diluted in equilibrium buffer were thawed, and to verify their integrity, sodium dodecyl sulfate–polyacrylamide gel electrophoresis (SDS–PAGE) was performed under denaturing and nondenaturing conditions (dithiothreitol (DTT) 1 mM) (Figure 2). To explore the possibility that purified IgE antibodies from an allergy patient cross‐recognize tumor antigens, we performed IHC using the IgE from the allergy patient as primary antibodies with the biopsies of the cancer patients. A very weak signal that was nonspecific in the 14 samples from the biopsy donor patients was detected, which could not be improved even by increasing the antibody concentration several times (Figure 3). However, we decided to purify IgE antibodies from each biopsy to perform IHC for the corresponding biopsy, and the result was a highly specific signal capable of recognizing tumor cells, without any reaction in the surrounding healthy tissue (Figure 4). To explore whether cross‐recognition among patients existed, IgE antibodies purified from the patients were tested by IHC with oncological tissue samples from the other donors. Regarding cross‐recognition, a positive reaction was not observed in any of the reactions carried out between the IgE antibodies of a patient with tissue from another donor.

Microscopically, a malignant epithelial neoplasm forms small nests at low and medium magnification in histological sections, with focally, tubular (glandular) structures in approximately 30% of neoplasms. The glands display an infiltrative pattern; they are surrounded by desmoplastic stroma. Neoplastic cells are cohesive, more or less featureless, cuboidal, or polygonal, with central nuclei that have mild to moderate atypia and amphophilic cytoplasm. Immunostaining with anti‐IgE antibody is positive, with moderate intensity and a diffuse and complete membranous pattern (surrounding the cells). In contrast, fibroblasts from the stroma surrounding the nidus and tubules were entirely negative on immunostaining (Figure 4). Unfortunately, in none of the histological sections was it possible to observe a positive signal showing the presence of eosinophils or mast cells in the inflammatory infiltrate of the oncological samples.

### 3.4 Quantification of serum cytokines

**FIGURE 5:**
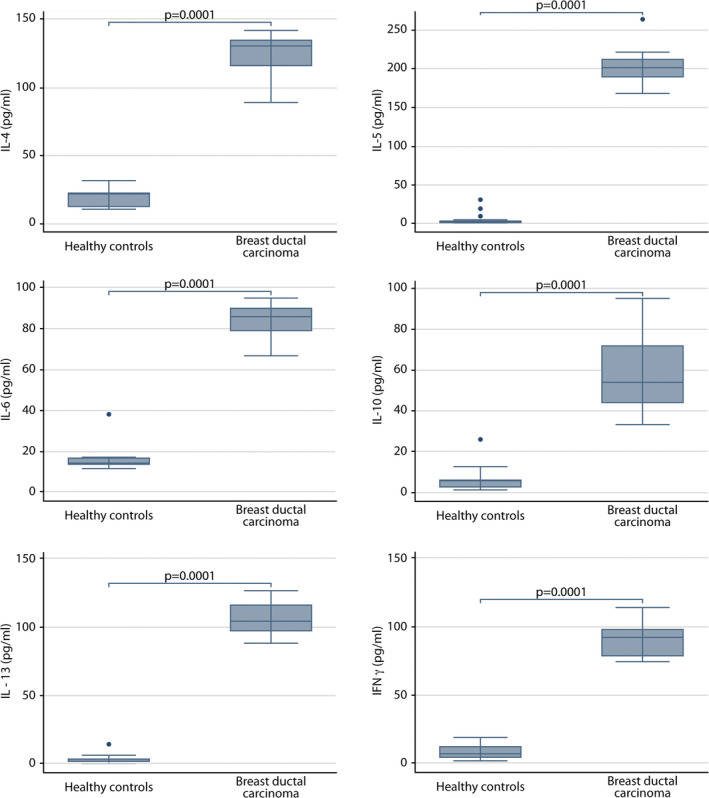
Comparison of serum cytokine concentrations between patients with confirmed diagnosis of ductal breast carcinoma (*n* = 14) and healthy controls (*n* = 17). The graph shows the median and IQR for each cytokine. The lines indicate significant differences with the corresponding *p* value.

Serum concentrations of all cytokines in breast ductal carcinoma donor patients were significantly higher than those in a group of healthy controls consisting of women of similar ages (59 years (49–65 years)). Notably, cytokines of the type 2 inflammatory response had the highest concentrations (IL‐4 (cancer patients: 130.5 pg/mL (116–135 pg/mL); healthy controls: 22.2 pg/mL (12.86–22.86 pg/mL)), IL‐5 (cancer patients: 202 pg/mL (191–213 pg/mL); healthy controls: 3.4 pg/mL (2.7–4.7 pg/mL)) and IL‐13 (cancer patients: 105.5 pg/mL (98–117 pg/mL); healthy controls: 2 pg/mL (1.4–3.6 pg/mL))) compared with cytokines representative of the type 1 inflammatory response (IL‐6 (cancer patients: 86 pg/mL (79–90 pg/mL); healthy controls: 14.3 pg/mL (13.6–16.7 pg/mL)); and INF‐γ (cancer patients: 93 pg/mL (79–99 pg/mL); healthy controls: 7 pg/mL (4.4–12 pg/mL)), and even the regulatory cytokine IL‐10 (cancer patients: 52 pg/mL (44–58 pg/mL); healthy controls: 6.3 pg/mL (2.5–6.3 pg/mL))) (Figure 5).

## 4 DISCUSSION

In this work, which was designed to explore the possibility that IgE recognizes specific antigens of tumor cells, we show that this antibody isotype can distinguish breast cancer malignant cells.

We previously explored the importance of the Th2 inflammatory response in developing tumor lesions using a murine model with parasitic infection to induce a Th2 inflammatory response. We observed that high concentrations of IgE antibodies, eosinophils, and neutrophils were related to the decrease in tumor lesions induced by a mutagen.^13^ With this background, we decided to explore the possibility that this inflammatory response might favor recognition of tumor cells in a clinical setting.

A new field of scientific research called allergo‐oncology is based on the clinical observation in which a possible inverse relationship between allergy history and absence in development of certain types of cancer has been described. This negative relationship has been particularly described with respect to childhood leukemia, glioma, pancreatic, neck, and head cancer.^9^, ^14^, ^15^, ^16^ It has been proposed that for cancer control, effector immunological phenomena such as degranulation of cells such as eosinophils, basophils, and mast cells, as well as recognition of antigens by IgE isotype antibodies occur in a patient with allergy^17^; however, the immunological mechanisms related to the allergy‐cancer relationship have not yet been clearly described.

In this work, we explored the possibility that IgE antibodies from allergy patients recognize antigens in breast ductal carcinoma. We found a positive signal, which was very weak even with high concentrations of IgE antibodies. Then, we tested the IgE‐breast cancer interaction using IgE purified from the tissue samples of each of the donor patient as the primary antibodies. In this case, we found positive immunostaining with moderate but highly specific intensity because they only recognized tumor cells without recognizing the healthy peripheral tissue surrounding the infiltrating neoplastic cells. The observation of positivity in all samples and the absence of cross‐reaction between the IgE antibodies of the patients and tumor tissues of the other patients suggests that the immunological response that induces production of IgE antibodies to the tumor antigens is specific to each individual. Although the affinity chromatography method used for the purification of IgE antibodies is highly specific, as shown in the images of the electrophoresis with IgE, where the main band of 188 kDa can be seen,^18^, ^19^, ^20^ in addition to other bands probably a product of the interactions between the chains that make up the immunoglobulin; contamination or subsequent degradation cannot be ruled out, even to a greater extent in IgE, an antibody with the shortest half‐life.^21^ However, the appropriate selection of a specific secondary antibody decreased the effect of these adverse conditions on the IHC result.

IgE is a predominant antibody isotype in the allergy response and the inflammatory response to helminth parasites; however, in recent years, evidence indicating that these antibodies play an important role in antitumor immunosurveillance has increased.^22^

A significant step forward in the field of allergo‐oncology is the fact that an anti‐folate receptor alpha IgE antibody, which in clinical phase 1 has shown a series of characteristics typical of this isotype,^23^ already exists, demonstrating safety with an administered dose in the range of 70 μg–12 mg and antitumor activity in patients with solid tumors.^24^ However, the fact that IgE immunoglobulins naturally recognize tumor antigens in patient samples, as demonstrated by our main finding in this work, might be the first step for future projects focused on exploring these antibodies for possible treatments.

On the other hand, this finding may relate to spontaneous tumor regression in breast cancer, a rare and unexpected phenomenon in the medical literature; however, it is likely.^25^, ^26^ Regarding this phenomenon, Boier et al. document that the type 2 inflammatory response directly blocks spontaneous mammary carcinogenesis by inducing terminal differentiation of cancer cells. The Th2 response, as mainly represented by thymic stromal lymphopoietin (TSLP), causes epigenetic reprogramming of tumor cells, activating mammary gland differentiation and suppressing the epithelial‐mesenchymal transition.^27^

Her‐2‐neu is a protein that participates in normal cell development. Some types of cancer cells, such as breast, ovarian, bladder, pancreas, stomach, and esophageal cancers, produce abnormal amounts of HER2/neu. However, more than being a prognostic marker, determination of this molecule serves as a diagnostic guide, given that some treatments are directed toward Her‐2‐neu as a therapeutic target.^28^ Ki67 is a cell proliferation marker with expression in breast tumors that has been related to worse prognosis and good response to chemotherapy treatment.^29^ On the other hand, progesterone and estrogen receptors are very useful histological biomarkers to guide prognosis and treatment in breast cancer, especially hormone‐positive cases.^30^ In this work, it was not possible to observe a characteristic pattern of intensity that could be associated with the positive signal when exposing tumor tissues to IgE antibodies; however, we cannot rule out this possibility. This should be addressed in future studies that include a greater number of oncological patients.

To explore the immune response in the biopsy donor patients in this work, the cytokines IL‐4, IL‐5 and IL‐13 were quantified along with IL‐6, INF‐γ and IL‐10. In all cases, the cytokines were present at higher levels in the cancer patients than in the healthy women of the control group. Notably, serum levels of cytokines representative of the type 2 inflammatory response were higher, a finding without precedent in the oncological literature; however, this is a prerequisite to the IgE‐inducing response, even though serum levels were higher than those reported in patients allergic asthma.^31^ On the other hand, the immunoglobulin levels were very similar to those reported by Ali et al. for the initial stages of breast cancer,^32^ except for IgE, which is not usually reported because it is not considered related to the immune response against this type of tumor.

Th2 cytokines are related to increased levels of serum IgE, which would favor the interaction of this immunoglobulin isotype with tumor antigens; however, the relationship of this inflammatory response with the different types of tumors is still controversial. There are four hypotheses that may explain the complex relationship between the type 2 inflammatory response and development of cancer: (A) Th2 chronic inflammatory response induces constant infiltration of inflammatory cells into tissues, remodeling and activation of enzymes with cytotoxic results, therefore the risk of developing malignancy increases^33^; (B) allergies are a reflection of general immune hyperreactivity, which improves immunosurveillance and triggers more intense responses against various antigens, probably tumors, which decreases the risk of developing cellular malignancy^33^; (C) physical effects of allergies such as coughing or sneezing may act as cleansing mechanisms to remove potentially toxic, mutagenic, or carcinogenic toxins from the respiratory tract, thereby reducing the risk of accumulation and development of cancer^34^; and finally, (D) the type 2 inflammatory response polarizes the immunological mechanisms, which causes shortages of others such as production of IgG antibodies or CD8 T lymphocytes, which would favor a lack of protection against malignant cells.^33^ These hypotheses show that the relationship between the Th2 inflammatory response and cancer development is much more complex than merely a statistical association.

Regarding the probable mechanism involved in the activity of the Th2 response and its effect on cancer development, Neuchrist et al. found that IgE is the antibody with the highest serum presence in patients with head and neck cancer.^35^ On the other hand, it has been documented that IgE in pancreatic cancer patients induces antibody‐dependent cellular cytotoxicity.^36^ Regarding the latter, the presence of eosinophils in the blood and infiltrates around tumor tissue has been associated with favorable prognosis, particularly in patients with solid tumors.^37^, ^38^

The immune system has evolved to protect the host from a constantly evolving universe of risks, many continually evolving in parallel. For correct elimination of any threat, identification mechanisms, either innate or adaptive, and effector mechanisms must be activated depending on the location and nature of the identified antigen.^39^ In this work, we demonstrated that IgE antibodies are capable of identifying antigens in tumor cells; when performing specific immunostaining to identify eosinophils or mast cells infiltrated in tumor tissue, none of these effector cells of the Th2 inflammatory response were found.

This study has some limitations. We consider that the most important is the number of patients recruited and the impossibility of carrying out in vitro studies to explore activation of effector cells. Both limitations are related to the limited amount of purified antibodies from each patient due to the clinical condition of the patients, with therapeutic attention to their pathology being prioritized. This exploratory study allows for future explorations of the effector mechanisms involved in antitumor defense that have not yet been contemplated.

Together, this information allows us to hypothesize that the immune system can eliminate tumor cells previously identified by IgE antibodies; however, a response lacking the appropriate effector cell infiltrate would allow for tumor development. This hypothesis opens the possibility of new explorations into the pathogenesis of cancer development, especially of solid tumors.

## AUTHOR CONTRIBUTIONS

**Espiridión Ramos‐Martinez:** Conceptualization (lead); data curation (equal); formal analysis (equal); investigation (equal); methodology (equal); writing – original draft (equal). **Francisco Javier García‐Vazquez:** Investigation (equal); methodology (equal). **Ramcés Falfán‐Valencia:** Investigation (equal); validation (equal); writing – review and editing (equal). **Jorge Rojas‐Serrano:** Formal analysis (equal); investigation (equal); methodology (equal); validation (equal); writing – review and editing (equal). **Ana Alfaro‐Cruz:** Investigation (equal); methodology (equal); validation (equal); visualization (equal). **Marcela Pérez‐Villaseñor:** Investigation (equal); methodology (equal); validation (equal); visualization (equal). **Gerardo Aristi‐Urista:** Investigation (equal); methodology (equal); validation (equal). **Jesús Pérez‐Hernández:** Methodology (equal). **Rosario López‐Vancell:** Investigation (equal); resources (equal). **Andrea Velasco‐Medina:** Conceptualization (equal); investigation (equal); methodology (equal); validation (equal); visualization (equal). **Guillermo Velázquez‐Sámano:** Investigation (equal); methodology (equal); project administration (equal); resources (equal).

## FUNDING INFORMATION

This work was performed with a research grant from the Hospital General de México “Dr. Eduardo Liceaga”, number of protocol: DI/19/309‐A/04/102; and the PAPIIT project, IN216720.

## CONFLICT OF INTEREST STATEMENT

All authors declare no conflict of interest.

## ETHICS STATEMENT

The study was conducted in full compliance with the Declaration of Helsinki. The institutional ethics committee approved the study protocol (approbation number: DI/19/309‐A/04/102). The participants, or their legal representatives, gave their written informed consent.

## INSTITUTIONAL REVIEW BOARD STATEMENT

The study was conducted according to the guidelines of the Declaration of Helsinki and approved by the Institutional Review Board (Mexico City, Hospital General de México, DI/19/309‐A/04/102).

## INFORMED CONSENT STATEMENT

Informed consent was obtained from all subjects involved in the study.

## Data Availability

Authors confirm the raw data to support this study's conclusions are included in the manuscript. The corresponding author will provide more information, upon reasonable request, to any qualified researcher.
